# Crowned Dens Syndrome: A Case Report and Literature Review

**DOI:** 10.3389/fmed.2021.528663

**Published:** 2022-01-24

**Authors:** Pan Huang, Min Xu, Xiao-Ying He

**Affiliations:** ^1^Department of Neurology, People's Hospital of Deyang City, Sichuan, China; ^2^Department of Neurology, The Second People's Hospital of Deyang City, Sichuan, China; ^3^Department of Neurology, The Affiliated Hospital of Southwest Medical University, Sichuan, China

**Keywords:** crowned dens syndrome, disease diagnosis, neck pain, odontoid syndrome, case report, literature review

## Abstract

Crowned Dens Syndrome (CDS) is the leading cause of acute neck pain and a major cause of reduced neck mobility. The diagnosis of CDS is challenging as clinicians currently have limited awareness of the symptoms of the disease. In this article, we report a case of odontoid syndrome as a reference for the diagnosis and treatment of CDS.

## Introduction

Crowned dens syndrome (CDS) refers to the findings of a crown-like odontoid in radiographic images (1). The typical clinical manifestations of CDS include acute neck pain accompanied by stiffness and limited mobility, fever and increased erythrocyte sedimentation rate (ESR) and/or C-reactive protein (CRP) levels. Imaging indicates that high-density, irregular calcium deposits of varying dimensions are formed around the odontoid process but mostly on the posterior surface of the odontoid process (2). Acute neck pain may be associated with the deposition of hydroxyapatite or calcium dicalcium pyrophosphate crystals in the ligaments around the odontoid. In imaging, the deposited crystals produce a crown or halo-like feature around the odontoid process (3). In this study, we report a case of CDS that can help to improve clinical understanding of the condition.

A 73 year-old female presented with head and neck pain that occurred during sitting (at ~9:00 on January 3rd, 2019). The pain was persistent and severe but tolerable. The pain increased significantly when the patient turned her head and was accompanied by neck stiffness. The patient did not report difficulties when speaking, numbness, chills, fever, abnormal bowel movements, breathing difficulties or reduced vision. The patient thought that the condition was due to cervical spondylosis and received “massage and topical plaster” treatment in the clinic but the symptoms did not improve significantly. The patient began to experience persistent pain in the scalp on both sides of the temporal roof. The patient sought diagnosis and treatment and was admitted to our hospital and clinic diagnosed with head and neck pain.

**Figure 1:**
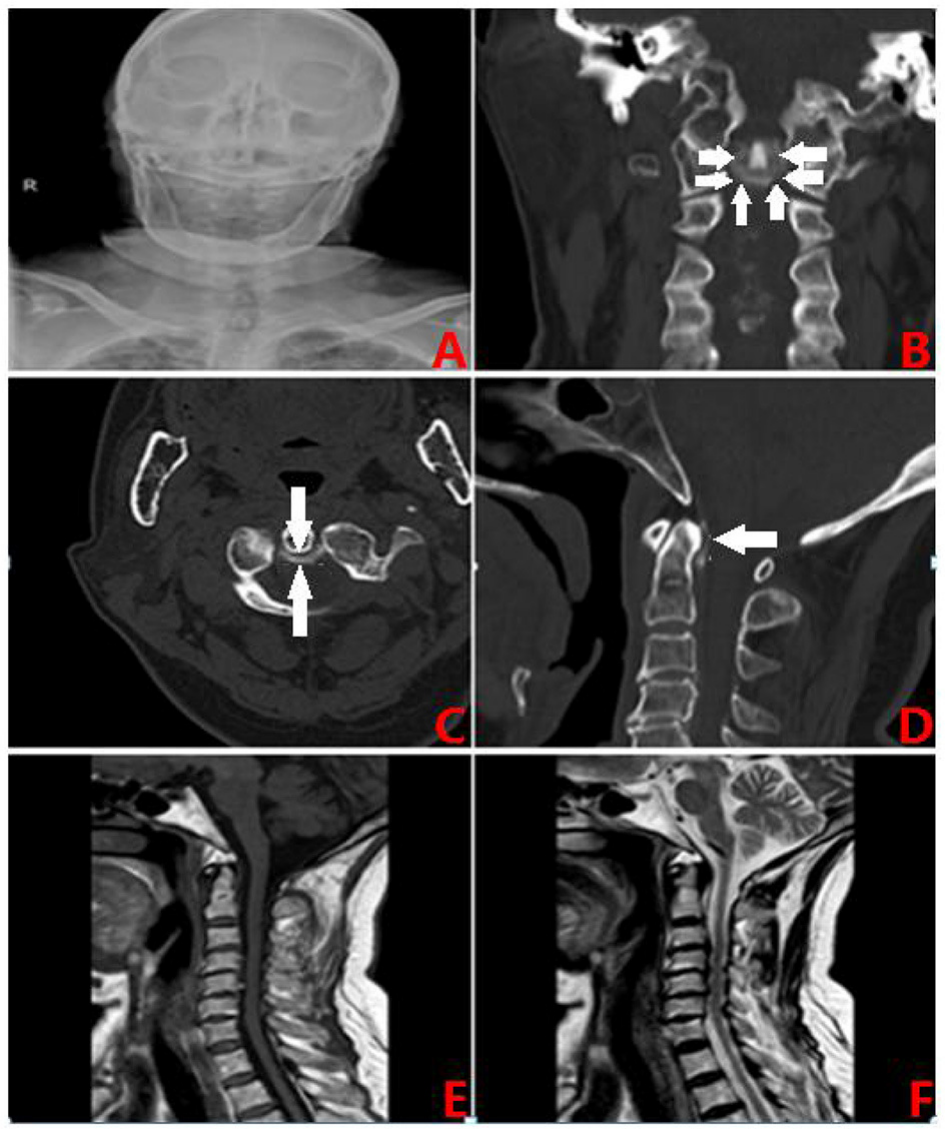
**(A)** The X-slice. **(B)** CT coronal section: crown sign. **(C)** CT transverse position: ring high. **(D)** CT Median sagittal section: linear calcification. **(E)** MRI T1 phase. **(F)** MRI T2 phase.

The patient had a 20-year history of type 2 diabetes (T_2_DM) and was using biosynthetic human insulin injections to control her blood glucose levels, however, the levels were not regularly monitored. Four years earlier, the patient was diagnosed with coronary atherosclerotic heart disease and subsequently received a stent. Postoperatively, the patient was prescribed aspirin (0.1 g qd) and clopidogrel (75 mg qd) to prevent platelet aggregation which was stopped after 1 year. The patient claimed to have no history of smoking, drinking, or any genetic diseases. She was admitted to hospital for physical examination and had a body temperature of 36.5°C, a pulse of 80 beats/min, a respiratory rate of 20 breaths/min and blood pressure of 120/77 mm/hg. Cardiopulmonary abdominal examination showed no apparent abnormalities. The patient was conscious and had no obvious facial and tongue paralysis but had restricted neck turning and tenderness in the back of the neck. The muscle strength of the limbs was grade five and muscular tension was normal. The somatosensory examination was normal and bilateral pathological signs and meningeal irritation signs were negative. The pain visual analog score was seven points. Cervical spondylosis was suspected and a cervical X-ray showed that the atlantoaxial joint space was wide on the left side and narrow on the right side (Figure 1A). The cervical vertebra CT three-dimensional (3D CT) images showed high-density shadows of the atlantoaxial joint in the coronal, transverse sections (Figures 1B,C) and median sagittal sections suggesting linear calcification (Figure 1D). Cervical MRI indicated C3/4, C4/5, C5/6 and C6/7 disc herniation, corresponding to spinal dural sac compression and spinal canal stenosis (Figures 1E,F). The patient's erythrocyte sedimentation rate was 43 mm/h (0–20 mm/h), the WBC was 9.8 × 10^9/*l*^ (0–10 × 10^9/*l*^) and the hs-CRP level was 56 mg/l (0.5–10 mg/l). These findings were consistentwith Crowned Dens Syndrome (CDS). After 1-week of neck rest fixation and non-steroidal anti-inflammatory drug (NSAID) administration, the patient's symptomsimproved and no pain was observed during outpatient follow-up after 2 weeks. The erythrocyte sedimentation rates also returnedto normal.

## Discussion

Currently, the number of cases of CDS reported in China and around the world is low (4, 5). Matsumura and colleagues (6) reported a single case in the New England Journal of Medicine, whilst Kuriyama and coworkers (7) reported a case in the Canadian Medical Journal. CDS is not uncommon but is often misdiagnosed or ignored due to a lack of knowledge about the disease. Statistics suggest that CDS is a major cause of acute neck pain with limited mobility that accounts for 2% of acute neck pain cases (8). This may be partly due to the variable presentation of CDS as demonstrated in our cases.

Regarding the pathogenesis of CDS, calcium crystals are deposited in the soft tissue around the odontoid leading to ossification of the posterior longitudinal ligaments of the cervical spine. The main cause of CDS is crystal deposition disease, including calcium hydroxyapatite and calcium pyrophosphate deposition diseases. Studies have shown that 51% (29/51) of the patients with calcification around the dentate process have CPPD deposition and 80% of cruciate ligament calcification is related to the deposition of CPPD crystals (9). CPPD is often deposited around the C1-C2 joints involving the odontoid process and surrounding ligaments amongst which calcifications of the cruciate ligament, ligament flavum, and transverse ligament of the atlas are more common. CPPD crystal deposition can cause focal aseptic inflammation to stimulate C1 or C2 nerve roots that in turn can cause pain when the neck is rotated (10–12). However, the reason why CPPD is deposited is not yet clear. It may be related to increased pyrophosphate levels in the joints caused by the increased decomposition of adenosine triphosphate and genetic factors (ANKH genes) (13).

Hydroxyapatite (HA) is the main component of human bone tissue. Studies have found that HA crystal deposits can trigger CDS but it is rare and only affects middle-aged women. The imaging findings of HA are similar to those of CPPD but the difference is that HA is often deposited on the anterior cervical muscle tendons or intervertebral disks, whereas CPPD is often deposited on the intervertebral disks, cervical ligamentum flavum, intervertebral joints, or transverse ligaments (14, 15). At present, most clinical case reports show that the probability of CDS in CPPD disease is higher than that in HA crystal deposition disease (16).

The incidence of CDS is significantly related to age and gender. In a retrospective study involving more than 2,000 patients with acute neck pain, 60% of CDS were elderly female patients (8). Neck CT scans in 554 outpatients found that 88 patients were confirmed to have CDS. Among these patients, the incidence of CDS was higher in women and increased significantly with age (17). Oka et al. studied 72 cases of CDS patients and found that CDS usually occurs in the elderly with an average age of 71.4 years and female patients accounting for 60% of cases (18). Also, genetic factors, trauma, inflammation and the existence of many diseases can affect the development of the course of CDS (16, 19).

The current diagnostic criteria for CDS are (20); (1) A history of acute neck pain and limited cervical activity, (2) Laboratory tests showing elevated inflammatory markers such as erythrocyte sedimentation rate, C-reactive protein and white blood cell ratios. Studies have shown that most patients with CDS have elevated inflammatory markers in serum laboratory tests with a detection rate of 88.3%. Around one-third of CDS cases have a fever and elevated ESR. Most patients can have elevated CRP and WB which can return to normal after routine anti-inflammatory treatments, (3) Deposition of calcium salts around the odontoid in cervical CT scans. Computed tomography CT scans of C1-C2 segments of the neck revealed that calcification deposits around the odontoid process are the “gold standard” for clinical diagnosis with a diagnosis rate of 97.1%. However, long-term follow-up of computed tomography CT scans has shown that the imaging manifestations of CDS may gradually disappear due to the absorption of calcifications. Also, the diagnostic function of X-rays and MRI in CDS is lower than that CT, and (4) Exclusion of other diseases that result in similar symptoms.

The treatment of CDS is relatively simple and includes NSAIDs or more effective short-course corticosteroids. The optimal treatment involves combined drug therapy that is effective but long-term use is not advised due to the possibility of side effects. However, Uh et al. found that some CDS patients have incomplete pain relief after using NSAIDs and glucocorticoids yet these can be alleviated by corticosteroid injections in C1-C2 lesions. These findings suggest that NSAIDS and corticosteroids cannot completely resolve CDS pain symptoms (11). Knier et al. found that for intractable CDS cases, colchicine can be used but it is less frequently used compared to glucocorticoids and NSAIDs and has a longer treatment course. It can be used when the other drugs are ineffective.

In summary, CDS is not uncommon in the clinic. However, due to an insufficient understanding at the primary level, diagnosis is often inaccurate and treatment is delayed. In the future, patients with acute neck pain and elevated inflammatory indices should be considered for CDS and CT scans of the cervical spine should be performed to improve the diagnosis of CDS.

## Ethics Statement

Written informed consent was obtained from the individual for the publication of any potentially identifiable images or data included in this article.

## Author Contributions

All authors listed have made a substantial, direct, and intellectual contribution to the work and approved it for publication.

## Conflict of Interest

The authors declare that the research was conducted in the absence of any commercial or financial relationships that could be construed as a potential conflict of interest.

## Publisher's Note

All claims expressed in this article are solely those of the authors and do not necessarily represent those of their affiliated organizations, or those of the publisher, the editors and the reviewers. Any product that may be evaluated in this article, or claim that may be made by its manufacturer, is not guaranteed or endorsed by the publisher.
